# In utero and childhood exposure to organochlorines and perfluorinated chemicals in relation to sperm aneuploidy in adulthood

**DOI:** 10.21203/rs.3.rs-8129828/v1

**Published:** 2025-12-05

**Authors:** Melissa J. Perry, Alessandra Meddis, Heather A. Young, C. Rebecca Robbins, Esben Budtz-Jørgensen, Niels Jørgensen, Jónrit Halling, Pál Weihe, Philippe Grandjean, Maria Skaalum Petersen

**Affiliations:** George Mason University; Copenhagen University; The George Washington University; The George Washington University; Copenhagen University; Copenhagen University Hospital – Rigshospitalet and International Centre for Research and Research Training in Endocrine Disruption of Male Reproduction and Child Health (EDMaRC); the National Hospital of the Faroe Islands; University of the Faroe Islands; University of Southern Denmark; the National Hospital of the Faroe Islands

## Abstract

**Background:**

Sperm chromosomal abnormalities are linked to infertility and may be caused by endocrine disrupting chemical exposures during development.

**Objectives:**

We examined whether exposure to organochlorine compounds (OC), including polychlorinated compounds (PCBs), and perfluorinated compounds (PFASs) measured repeatedly since birth predicted sperm chromosomal abnormalities in young adulthood.

**Methods:**

Aneuploidy was determined in semen samples obtained from 96 Faroese young men aged 22–24 years who were members of a birth cohort created in 1986–1987. Their current and previous serum as well as cord blood were analyzed for DDE, major PCB congeners (118, 138, 153, and 180), and PFAS (PFOA, PFOS, PFNA, PFDA, and PFHxS). Incidence rate ratios between the exposures and the risk of an extra sex chromosome in adult sperm were assessed as indication of meiotic errors. The mixture effect for overall exposures (PCBs and/or PFASs) was estimated as the change in the percentage of each type of disomy for a doubling of the exposures for two individuals within the same smoking status and abstinence time group.

**Results:**

Higher concentrations of organochlorines in cord blood and in serum at ages 7, 14 years and 22 years were associated with increased proportions of chromosomal disomies. The PCB concentration in cord blood was associated mainly with having an extra Y chromosome (p-value: 0.006), while PFAS concentrations at adulthood were consistently associated with XX18 and YY18 disomies (p-values < 0.05).

**Discussion:**

These findings provide new evidence that fetal and subsequent chemical exposures can have enduring influence into adulthood on the formation of male germ cells.

## Introduction

Male fecundity has been declining in Western countries, and analyses that show sperm counts during the last 50 years have decreased by approximately 50% (Carlsen et al., 1992; Levine et al., 2022). Additional studies suggest that testicular dysgenesis (TD) syndrome involves lowered sperm counts, increased urogenital abnormalities and testicular cancer (reviewed in Skakkebaek et al., 2016). These andrological indicators have direct bearing on male reproductive capacity, a matter of concern, as various countries are at or approaching total fertility rates below population replacement levels (Levine et al, 2017).

Chemical exposures to the fetal testis can cause TD in animals that may be passed on to subsequent generations (Guerrero-Bosagna and Skinner, 2009; reviewed in Bonde et al., 2016). The endocrine-disrupting effects of polychlorinated biphenyls (PCBs) and the pesticide metabolite, dichlorodiphenyldichloroethylene (p,p’-DDE), are of major concern because these organochlorine compounds (OCs) persist in the environment and are known to cause developmental impacts (Gore et al., 2015). While PCB usage has been phased out, the parent pesticide, dichlorodiphenyltrichloroethane (DDT), remains a choice for vector control in malaria-endemic countries. Similarly, PFAS exposures have changed over time, but early-life exposures may lead to lower sperm quality (Tarapore and Ouyang, 2021).

Fetal abnormalities in the chromosome count, known as aneuploidy, account for 50% of preterm pregnancy losses (Nagaoka et al, 2012), but the causes of aneuploidy during oogenesis and spermatogenesis are largely unknown. Errors in spermatogenesis are thought to account for 50% of Klinefelter (XXY) and Turner’s syndrome (45,X) cases (Hassold and Hunt, 2001), two genotypes that can occur in sperm and may lead to infertility. Data from the European birth registry EUROCAT have shown an increase in the prevalence of XXY, but not XYY (Morris et al., 2008). Given that XXY may originate from nondisjunction in the paternal sex chromosomes during spermatogenesis, this increase is suggestive of additional TD impacts on germ cell production and genetic integrity, thereby resulting in population level increases in paternally derived congenital abnormalities.

We have shown previously that adult *p,p’*-DDE and PCBs exposure levels are associated with increased occurrence of sperm sex chromosome disomy (McAuliffe at al., 2012a; Perry et al. 2016). A meta-analysis showed that prenatal *p,p’*-DDE exposure increased the overall risk of male reproductive disorders that included cryptorchidism, hypospadias, impaired semen quality and testicular cancer (Bonde et al., 2016). Further, increased occurrence of sperm disomy has been reported in males with increased PFAS exposures (Governini et al., 2015). However, it is unknown whether prenatal contaminants exposure is also associated with sperm chromosomal abnormalities in the offspring. The goal of the present study was to obtain evidence of human *in utero* and childhood exposure to the main organochlorines, i.e., *p,p’*-DDE and major PCB congeners, and PFASs, in regard to sperm aneuploidy in adulthood. Due to the wide range of contaminant exposures (Barr et al., 2006; Needham et al., 2011; Timmermann et al., 2019), the study was carried out in a previously established general population birth cohort at the Faroe Islands.

## Methods

### Study population and recruitment

The study population consisted of adult men at age 22–24 years who were members of a birth cohort (Cohort 1) created in 1986–1987; inclusion and exclusion criteria as well as recruitment practices have been outlined previously (Grandjean et al., 1992). For men in the birth cohort, the mothers were recruited before birth and provided a blood sample at week 34 of pregnancy and a cord blood sample was obtained at delivery. Participants were then followed prospectively, with data and blood samples gathered again at ages 7, 14, and 22 years. All male subjects were invited to participate in a study of semen quality and were examined in 2009–2010. All subjects and their mothers signed an informed consent form before participation. All procedures were approved by the Faroese ethical review committee and the corresponding Institutional Review committee in the U.S.

### Semen samples and analysis

Semen analysis methods have been described previously (Halling et al., 2013). Briefly, subsequent to the examination at age 22 years, the men produced a semen sample by masturbation in a private room near the laboratory. Abstinence time was recorded at the time of sample collection. The two technicians involved were trained at the semen laboratory at the National University Hospital (Rigshospitalet) in Denmark and conducted the semen analysis according to the World Health Organization (WHO) guidelines (WHO, 2010). For usage in sensitivity analyses of disomy data, semen values were dichotomized on the basis of reference values for three main semen quality parameters, i.e., sperm count (< 15 million sperm per milliliter of ejaculate), motility (< 40% total motile sperm), and morphology (< 4% normal forms) (WHO 2010).

### Sperm Disomy Analysis

To detect sex-chromosome disomy (aneuploidy involving an extra X or Y chromosome), a single investigator blinded to exposure status performed the assay by FISH analysis as described in detail previously (McAuliffe et al. 2012a). The FISH procedure was performed for three chromosomes of interest, X, Y, and 18 (autosomal control), with a series of nonoverlapping field images taken for each prepared FISH slide using a fluorescence laser scanning wide-field microscope. Sex-chromosome disomy was the primary outcome of interest because of its reproductive health impacts: *a*) it is the most frequent form of sperm aneuploidy and occurs twice as frequently as autosomal disomy; *b*) sperm that are disomic for X or Y are capable of fertilization; and *c*) sex-chromosome disomy results in viable offspring. The images were scored with Leica LASX software (version 3.1.5) designed to utilize scoring criteria for size and shape. A colocalization analysis allowed the software to identify sperm nuclei and the number of signals contained therein. The method has been shown to produce results quantitatively and qualitatively comparable to manual scoring (Perry et al., 2007, 2011, 2016).

### Exposure Analysis

A two-stage solid-phase extraction method followed by gas chromatography analysis with electron capture detection was used to quantify the four most prevalent PCB congeners, CB-138, CB-153, and CB-180, and the dioxin-like CB-118 (Grandjean et al. 1995; Petersen et al. 2006), along with *p,p′*-DDE. All serum results were adjusted for total serum lipid content and reported as micrograms per gram lipid. However, cord blood concentrations were expressed in terms of μg/mL whole blood (Grandjean et al. 2012). Lipid concentrations in cord blood are low and less variable than those in non-fasting postnatal serum. As lipid determination was not feasible for the material available, an average lipid concentration of 3 mg/mL was therefore used for converting the volume-based results into μg/g lipid (Grandjean et al., 2012). PCB exposure was also represented by the sum of serum concentrations of the most prevalent PCB congeners (ΣPCBs=(138 + 153 + 180)*2). The median limit of detection (LOD) was 0.03 μg/L, which, at a mean lipid concentration of 7.45 g/L, corresponds to 0.004 μg/g lipid. Nondetectable levels of PCB congeners and *p,p′*-DDE were assumed to be equal to 0.002 μg/g lipid, i.e., one-half the LOD. As a measure of overall PCB exposure, we used Σ3PCBs as the sum of the most prevalent congeners (138, 153 and 180) multiplied by 2 (Grandjean et al., 2008).

PFASs were likewise measured in whole blood from the cord and in serum on subsequent examinations, as previously described (Petersen et al., 2018; Timmermann et al., 2019). With a detection limit of 0.03 ng/mL, PFOS, PFOA, PFNA, PFDA and PFHxS were detectable in the majority of serum samples. In cord whole blood, only PFOS and PFOA were determined. With a detection limit of 0.03 ng/mL, samples with PFAS concentrations below that level were assumed to contain 0.015 ng/mL. The results were added to obtain the ΣPFAS concentration. The majority of measurements of PFNA, PFDA and PFHxS were undetectable in the cord blood sample, thus results for these exposures are shown only for samples at the older ages 7,14 and 22 years (adult).

### Statistical Analysis

Statistical analyses included analyses of blood samples from birth to adulthood in 96 men belonging to a Faroese birth cohort created in 1986–1987. The exposures to OCs and PFASs, were available at different time points across their lives (birth, 7, 14, and 22 years of age), while sperm was collected at adult age of 22–24 years. Pearson correlations were examined for exposures at each of the four time points and for different times for each of the exposures of interest. In particular, we provide results for PCB-118, PCB-138, PCB-153, PCB-180, Σ3PCBs, *p,p’*-DDE, PFOA, PFNA, PFDA, PFHxS, total PFOS, and ΣPFAS.

Descriptive statistics for demographic and semen parameters were summarized using frequency distributions, i.e., means and standard deviations or median and inter-quantile ranges. Poisson regression with robust standard errors was used to model the association between each of the disomy measures and organochlorine exposures (*p,p′*-DDE, PCBs, and PFASs) in adjusted analyses.

The number of disomic nuclei identified were summed for each subject and used as the unit of analysis. In the Poisson regression, the offset variable allows for control of time/space variation in the denominator. In this study, the source of variation referred to the number of nuclei scored per subject. The Poisson model was fitted using each disomy measure (XX18, YY18, XY18, or total sex-chromosome disomy) as the outcome variable, the natural logarithm of the number of nuclei as the offset variable, and the log2-transformed organochlorine and PFASs exposure of interest as the independent variable. One Poisson regression model was calculated for each time point of blood sampling for every disomy vs. exposure combination (PCB-118, PCB-138, PCB-153, PCB-180, Σ3PCBs, *p,p’*-DDE, PFOS, PFOA,PFNA, PFDA and PFHxS, and ΣPFASs). Covariates were identified based on *a priori* considerations (Blackwell and Zaneveld 1992; Hassan and Killick 2003; Vine 1996). Abstinence time (≤ 3 days, >3 days) and smoking status [ever vs. never] were included in the primary analysis (Model 1, M1). Results of M1 are then provided for the estimated relative change in the percentage of disomy at doubling of the exposure concentration for two men within the same smoking status and abstinence time group. This number was estimated as the exponential of the exposure regression coefficient. Because sperm concentration, motility, and morphology have been associated with disomy in prior studies (e.g., Martin et al. 2003; McAuliffe et al. 2012b; Vegetti et al. 2000), log-transformed sperm concentration, motility, and morphology were included as continuous variables for sensitivity analyses (Model 2, M2).

The quantile g-computation method was used to estimate the mixture effects in this sample of correlated multi-pollutants (Keil et al., 2020). This method fits a marginal structural model for all the exposures and provides results for the multi-pollutants mixture effect considering the sum of the regression coefficients for all exposures. A score test for the mixture effect is implemented to test whether the mixture effect (Ψ) is significantly different from 0. In this application, the exposures considered have well established deleterious health impacts as persistent pollutants, and a beneficial impact on spermatogenesis is biologically implausible and has not been previously documented. Therefore, we also implemented an extension of the quantile g-computation method with the restriction of non-negativity on the regression coefficients (unidirectionality assumption) and one-sided permutation test for non-null mixture effect.

The quantile g-computation (with and without restriction) was used to assess the mixture effect of individual PCBs and PFASs and for the mixture effect of PCBs and PFASs together. Moreover, when examining mixture effects for PCBs, we also adjusted for PFASs and, likewise, when considering the mixture effect of PFASs, we adjusted for PCBs. Given the skewed distributions, a log2 transformation was used for all exposures. A Poisson regression model included adjustment for smoking status and prior abstinence time in days, and a confidence interval were calculated with robust standard errors. Because exposures had similar scales, raw exposure values were included instead of quantiles. Results for the mixture effect are presented with estimates of exp(Ψ), which is interpreted as the estimated relative change in percentage of disomy if all exposure concentrations were doubled. In addition, we provide the corresponding *p*-value for independence between mixture and outcome (H_0_: Ψ = 0).

Complete case analysis was used for the models, and observations with missing data were automatically excluded. A *p*-value ≤ 0.05 was considered statistically significant. We used R software, for all data analyses.

## Results

Table 1 details the demographic characteristics of the study cohort. The men had a mean age of 23 years (median: 23.6; range: 22–24) and a mean BMI of 24.8kg/m2 (median: 23.8; range: 21.93–36.56). Over half of the men (54%) had smoked. The median sperm concentration was 47 million/mL, and 15% (n = 15) had sperm concentrations < 15 million/mL; 4% (n = 4) had < 40% progressively motile sperm, and 22% (n = 22) had < 4% normally shaped sperm. The average number of scored sperm nuclei was 9,450 (median 8021, range: 1212–32671).

Table 2 summarizes the distribution of lipid-adjusted *p,p’*-DDE and the major PCB and PFAS concentrations. Medians for *p,p’*-DDE were 0.6 μg/mL in cord blood and 0.2 μg/g at age 22. The cord blood, age 7, 14 and 22 medians for Σ3PCBs were 1.3 μg/mL, 2.4 μg/g, 1.0 μg/g and 0.7 μg/g respectively. The cord blood, age 7, 14, and 22 medians for PFOA were 2 μg/mL, 5 μg/g, 4.7 μg/g and 3.1 μg/g. P,p’-DDE, Σ3PCBs, PFOA and total PFOS all showed discernable decreases between ages 7 and 22.

**Figure 1.**
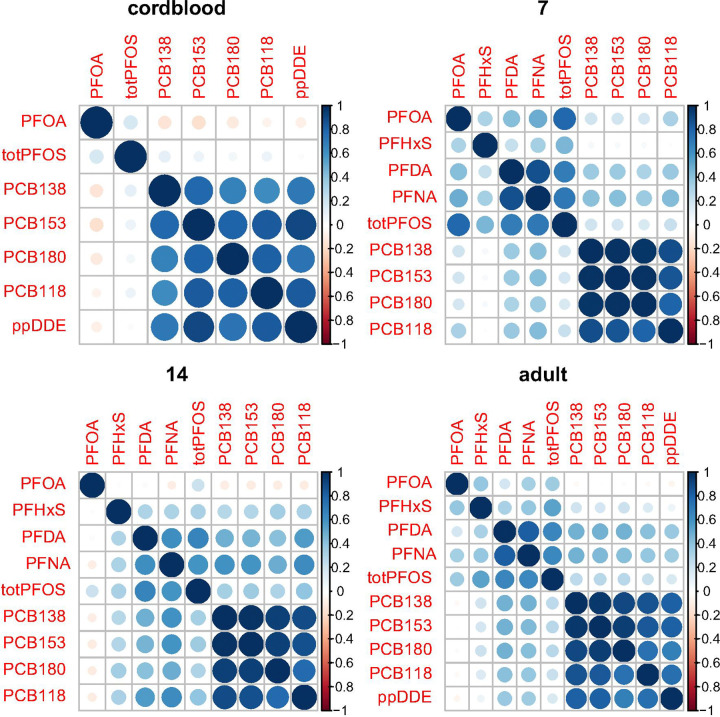
Pearson correlations among PCB and PFAS concentrations at four time points in the cohort. For cord blood, PFDA, PFNA and PFHxS are omitted because most measurements were below the detection limit. ppDDE is not shown for ages 7 and 14 years because those measurements were unavailable.

**Figure 2.**
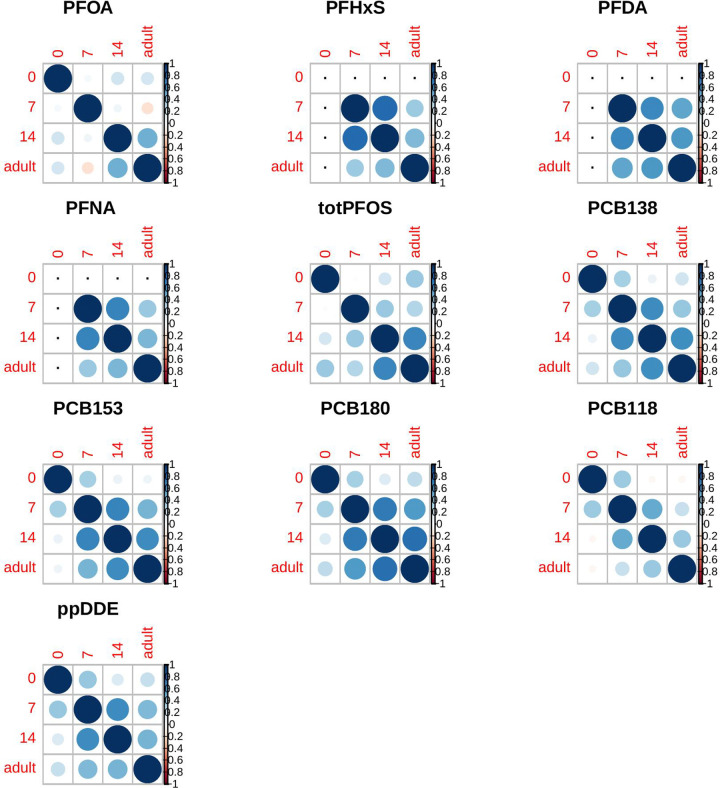
Pearson correlations of PCB and PFAS concentrations over time; missing correlations are marked with a black dot.

Individual PCB values were moderately to highly correlated across time, with the exception of measurements in cord blood (Fig. 1). Among PFAS values, PFOA showed the weakest correlation across time, having only a weak correlation between age 14 and adult (Fig. 1). In each sample (cord blood, age 7, age 14, and adult) PCBs were highly correlated (Fig. 2) among each other. A weaker correlation was seen for PFASs, where the strongest linear correlation was seen between PFDA and PFNA and total PFOS. A moderate correlation was seen between PFDA and PFNA with PCBs at older ages (age 14 and adult).

Results for single exposures are shown in Table 3. They represent the estimated relative change in the percentage of each disomy at a doubling of the exposure concentration for two individuals within the same smoking status and abstinence time group. This single-exposure analysis showed a significant association in cord blood between PCB138 and an increase of total disomy (1.166; 95% CI:1.055, 1.288), XX18 (1.148; 95% CI: 1.002,1.314), XY18 (1.129; 95% CI: 1.023,1.268) and YY18 (1.232; 95% CI: 1.057,1.436). However, this association remained significant only for XX18 at age 7 (1.361; 95% CI: 1.050,1.766) and no significant association was found at the older ages. Similar results were found for PCB153, where the analysis showed a significant association in cord blood with XY18 (1.245; 95% CI: 1.033,1.501) and total disomy (1.150; 95% CI: 1.019,1.298), but this association was not significant at older ages., and none of the associations showed significance at adult age.

The analysis for single PFAS exposures showed significant associations at age 7 between PFOA and an increase of YY18 (2.643; 95% CI: 1.153,6.058), PFNA and increase with all types of disomies (total disomy:1.581; 95% CI: 1.190,2.100) and PFHxS with total disomy (1.335; 95% CI: 1.162,1.534). At age 14, PFNA showed a significant association with XX18 (1.574; 95% CI: 1.082,2.290) and XY18 (1.492; 95% CI: 1.010,2.205); similar results were found for PFHxA. These PFAS components showed a significant association also at adulthood (Table 4).

Results for the sensitivity analysis where log-transformed sperm concentration, motility and morphology were also included in the model showed similar results to the single exposure model adjusting for abstinence time and smoking, with a significant association in cord blood between PCB138 and XY18, YY18 disomies and a significant association at age 7 and 14 for all PCBs components with an increase of XX18 disomy. No significant associations were seen at adulthood. For PFASs, the associations between PFNA and PFHxS with XX18 disomy were significant at age 7 and 14, and only the association with PFHxS remained significant at age 22.

Because of the correlations among PCBs and PFASs, we used quantile g-computation to assess mixture effects on the different disomies at each age. A Poisson regression model with robust standard errors was considered for each mixture of exposures. Results of the g-computation are provided in Table 5. When assessing the association for the PCB mixture, we included PCB180, PCB138 and PCB153. Results showed a significant association in cord blood with YY18 (p-value = 0.006) and total disomy (p-value = 0.009). Significant results were obtained at age 7 with the PCBs mixture and XX18 (p-value: 0.018) and at adult age with total disomy (p-value = 0.043). The mixture of PFASs, included PFOA, PFOS, PFNA, PFDA and PFHxS, with the exception in cord blood where only PFOS and PFOA were analyzed. Here, a significant association was found at age 7 with YY18 (p-value = 0.015) and at adult age with most of the disomies (p-value for XX18 = 0.025). When looking for the mixture effect of PCBs and PFASs together, a significant association was found at age 7 with total disomy (p-value = 0.007) and at adult age with XX18 (p-value = 0.008), XY18 (p-value = 0.013) and total disomy (p-value = 0.009).

When implementing the quantile g-computation with the unidirectionality assumption (non-negativity restriction), similar results were found with additional significant associations for the PCB mixture in cord blood with XX18 and XY18 and for the PFASs at age 7 with total disomy (p-value = 0.037) (Supplemental Table 1). We also considered the mixture effect of PCBs while additionally accounting for PFASs. In this analysis, the PCB mixture effect was significant in cord blood for YY18 (p-value = 0.036) and total disomy (p-value = 0.018) and at age 7 for XX18 (p-value = 0.006). Further, the mixture effect of PFASs adjusting on PCBs showed a significant association only at adult age, with XX18. However, significant results were found for association of a mixture effects including all PFASs and PCBs in cord blood with XX18 (p-value = 0.039); at age 7 with total disomy (p-value = 0.031) and at adult age with XX18 (p-value = 0.005), XY18 (p-value = 0.04) and total disomy (p-value: 0.028).

## Discussion

The present study showed an association between chromosome disomies in sperm from young adults and their present and past exposures to PCBs and PFASs. Because of some multicollinearity among these two types of environmental pollutants, identifying the unique source of the association is challenging. We observed that PCB mixtures have a significant association when evaluated in cord blood, particularly for YY18 and total disomy, and at age 7 with XX18. In contrast, PFAS exposures were mostly associated with chromosome disomies when measured in adulthood.

As already reported in our previous study (Perry et al., 2016), the men in this study had high levels of OC exposures *in utero*, presumably due to a maternal diet of contaminated seafood (Barr et al., 2006). PCBs *in utero*, at ages 7, 14, and 22 years were associated with an increased rate of XX, YY, and XY disomy, with age 7 and *in utero* PCB exposure showing stronger associations than exposure levels measured as an adult. p,p’-DDE showed a similar pattern of associations, though less strongly than PCBs. OC exposures were the highest at age 7, as previously reported (Barr et al., 2006). In addition to transfer via breast milk, reasons for this may include a lower relative blood volume and differential distribution compared to age 14, low consumption of whale meat and blubber in later childhood, and the potential impacts of whale meat and blubber dietary advisories first issued in 1998. Our results show that these associations are independent of simultaneous PFAS exposures. In addition, the results show an independent effect of PFASs, particularly at age 7 years, which likely includes exposures transferred via human milk (Morgensen et al., 2015).

Infertility affects approximately 8–12% of couples globally. Aneuploidy is the most common chromosomal anomaly in humans and is the leading genetic cause of miscarriage and congenital birth defects. Most aneuploidy originates from errors during oogenesis (Nagaoka et al, 2012), and evidence from *in vitro* fertilization (IVF) clinics shows aneuploid oocytes are the most common cause of IVF failures (Fragouli et al., 2008). However, non-disjunction of the sex chromosomes during spermatogenesis contributes to X- and Y- linked conditions in offspring, including Klinefelter and Turner Syndromes. The fundamental molecular mechanism(s) that causes non-disjunction in oocytes or spermatocytes has yet to be defined. Just as oocyte aneuploidy is thought not to be due to a single causal factor but to multiple effects that begin *in utero* and continue through a woman’s reproductive years (Nagaoka et al., 2012), spermatocyte aneuploidy is likely due to multiple etiologies. Based on the convergence of evidence from multiple animal and human studies, exposure to endocrine disrupting chemicals, including PCBs and PFASs, in early life is likely an important contributor to the disomies observed.

Animal studies have shown that prenatal and perinatal exposure of mice to PCBs at certain doses designed to simulate human exposure caused lasting reproductive toxicity, including morphological and functional sperm damage that were transmitted to at least two subsequent generations (Pocar et al., 2012). Reduced sperm concentration and abnormal growth of accessory sex organs has been shown in rodents as a result of *in utero* exposure to PCB 118 (Kuriyama 2004). A recent study in the Sprague-Dawley rats demonstrated that early-life organochlorine exposure harmed male reproductive capacity across multiple generations (Lessard et al., 2019). F_0_ females were exposed to a mixture of persistent organic pollutants (POPs), primarily organochlorines. *In utero* POPs altered sperm parameters in F_1_. Paternal exposure to POPs reduced semen quality in F_2_ males. Importantly, F_3_ males had the poorest pregnancy outcomes and generated the embryos with the greatest differential gene expression.

Increased maternal serum PCB concentrations are related to decreased birth weight in newborn boys, shorter gestation in girls, and smaller head circumference in both sexes (Hertz-Picciotto et al., 2005). Maternal OC exposures are associated with changes in the sex hormones of their sons, specifically *p,p’*-DDE and *p,p’*-DDT are inversely associated with luteinizing hormone and testosterone, and PCBs are associated with increased follicle stimulating hormone (Eskenazi et al., 2017).

Similar associations have been observed in epidemiologic studies between maternal serum *p,p’*-DDE concentrations and increased risk of developmental abnormalities in male reproductive organs (Longnecker et al., 2002). Prospective studies in humans have found strong associations between fetal PCB and *p,p’*-DDE exposure and congenital cryptorchidism (Brucker-Davis 2008). Other studies have found that males exposed *in utero* to diethylstilbestrol (DES) had increased prevalence of urogenital abnormalities (Palmer et al., 2009). Observational studies have shown inverse associations between PCBs and sperm motility among US men attending infertility clinics (Meeker et al., 2010) and positive associations between PCBs and abnormal morphology among US community recruited men (Mumford et al., 2015). Our prior studies have shown that semen quality among the Faroese men were at the same low level as reported for Danish men, and reproductive hormone levels indicated a lower Leydig cell capacity for testosterone production (Halling et al., 2013; Petersen et al. 2015). However, the relative role that pre- or postnatal OC exposures may be contributing to these changes is unclear (Petersen et al., 2018) and deserve further attention.

Our previous study showed that elevated exposures to *p,p’*-DDE and PCBs were significantly associated with increased rates of XX18, XY18, and total disomy in adult Faroes men (Perry et al. 2016). A sample of 50 men from Yucheng in China, who experienced severely elevated PCB exposure from ingesting contaminated rice oil, showed compromised sperm morphology and some association with increased sperm X/Y ratio, but there was no apparent association with sperm aneuploidy. This may be attributable to small and heterogenous sample size, delayed timing of exposure assessment, and/or an inability to adjust for relevant confounders.

While the Faroe Islands have been an important setting for understanding the transgenerational health impacts of persistent organic pollutants, our study was conducted in an ethnically and racially homogenous population, and generalizability to other populations may be an important consideration (Grandjean et al., 1992). We have previously reported associations between these same OC forms and sperm aneuploidy among men attending a US fertility clinic. The sample largely consisted of white urban Americans, with *p,p’*-DDE and PCB levels considerably lower than in the Faroes, and on par with those found in the US general population (McAuliffe et al. 2012a).

To our knowledge, this is the first human study suggesting *in utero* effects of OCs on testicular function specific to risk for producing chromosomally abnormal sperm in adulthood. While causality cannot be proven from this prospective study alone, the consistent and significant dose response relationships seen across disomy types lend strong evidence to the fetal origins of this expression of testicular dysgenesis and suggest that sperm aneuploidy is another outcome of the syndrome caused by early-life exposure to environmental endocrine disruptors.

These results provide important new evidence demonstrating how organochlorines can affect the function of the human fetal testis into adulthood. The increased rates of disomy among adult men who had higher organochlorine concentrations in their umbilical cord at birth suggests lingering impacts on the earliest and most fundamental stages of germ cell formation, when chromosomes are disjoined during meiosis. This increased risk of sperm aneuploidy can be added to the other proposed outcomes caused by testicular dysgenesis syndrome and may well become another outcome parameter that is sensitive to chemical insult to the fetal testis.

## Supplementary Material

Supplementary Files

This is a list of supplementary files associated with this preprint. Click to download.

• SupplementaryTable1.docx

## Figures and Tables

**Table 1:** Individual characteristics of study cohort of Faroese men (n = 96) at time of semen collection for tertile groups of Σ3PCBs.^a^

Variable	Level	low (n = 32)	medium (n = 31)	high (n = 33)	Total (n = 96)
Age (years)	median [iqr]	23 [22, 23]	23 [22, 23]	23 [22, 23]	23 [22, 23]
	Missing	0	1	1	2
Days of abstinence	> 3	21 (67.7)	18 (60.0)	21 (67.7)	60 (65.2)
n (%)	<=3	10 (32.3)	12 (40.0)	10 (32.3)	32 (34.8)
	Missing	1	1	2	4
Smoking ever	Yes	15 (46.9)	17 (56.7)	19 (59.4)	51 (54.3)
n (%)	No	17 (53.1)	13 (43.3)	13 (40.6)	43 (45.7)
	Missing	0	1	1	2
BMI (kg/m^2^)	median [iqr]	24.7 [21.6, 29.4]	23.4 [22.0, 25.4]	24 [21.8, 25.0]	23.9 [21.8, 26.3]
	Missing	1	0	1	2
Progressive motility (%)	median [iqr]	58 [52.0, 60.2]	59.2 [55.9, 64.0]	57 [55.0, 59.5]	58 [55, 62]
	Missing	0	1	2	3
Sperm concentration (10^6^/ml)	median [iqr]	38.2 [23.4, 82.7]	53.6 [35.3, 94.3]	44.6 [22.6, 59.9]	47 [23.8, 79.8]
	missing	0	1	1	2
Normal morphology (%)	median [iqr]	4.8 [ 3.4, 10.9]	6.5 [ 3.5, 10.2]	7 [ 4, 11]	5 [ 3.5, 10.9]
	Missing	0	0	2	2
Number of nuclei (103)	median [iqr]	7.6 [ 4.5, 10.7]	7.6 [4.6, 14.6]	9.1 [ 5.9, 12.0]	8.0 [4.8, 11.7]

a Σ3PCBs =(PCB138 + PCB153 + PCB180)*2.

**Table 2:** Distribution of PCBs and PFASs over time in study cohort of Faroese men (n = 96).^a^

	Level	Birth (μg/mL)^b^	Age 7 (μg/g)^c^	Age 14 (μg/g)	Age 22 (μg/g)
PCBs					
118	median [iqr]	0.1 [0.0, 0.1]	0.1 [0.0, 0.1]	0 [0.0, 0.1]	0 [0, 0]
	Missing	3	50	20	0
138	median [iqr]	0.2 [0.1, 0.3]	0.4 [0.2, 0.5]	0.1 [0.0, 0.1]	0.1 [0.1, 0.2]
	Missing	3	50	18	0
153	median [iqr]	0.3 [0.2, 0.5]	0.5 [0.2, 0.6]	0.2 [0.1, 0.4]	0.1 [0.1, 0.2]
	Missing	3	50	18	0
180	median [iqr]	0.1 [0.1, 0.3]	0.3 [0.1, 0.4]	0.2 [0.1, 0.3]	0.1 [0.0, 0.2]
	Missing	3	50	18	0
p,p´-DDE	median [iqr]	0.6 [0.3, 1.1]	0.9 [0.7, 1.6]	0.7 [0.4, 1.1]	0.2 [0.1, 0.3]
	Missing	3	24	20	0
Σ3PCBs^d^	median [iqr]	1.3 [0.7, 2.3]	2.4 [1.1, 3.1]	1 [0.6, 1.7]	0.7 [0.4, 1.1]
	Missing	3	50	18	0
PFASs					
PFOA	median [iqr]	2 [1.1,3.6]	5 [3.9, 6.5]	4.7 [3.8, 5.8]	3.1 [2.3, 4.2]
	Missing	7	21	20	0
PFNA	median [iqr]	-	0.7 [0.6, 0.9]	0.7 [0.5, 0.9]	1 [0.8, 1.2]
	Missing	96	21	20	0
PFHxS	median [iqr]	-	0.9 [0.7, 1.2]	0.7 [0.5, 0.8]	0.7 [0.5, 0.8]
	Missing	96	21	20	0
PFDA	median [iqr]	-	0.2 [0.2, 0.3]	0.3 [0.2, 0.4]	0.4 [0.3, 0.5]
	Missing	96	21	20	0
Total PFOS^e^	median [iqr]	2.3 [2.0, 3.1]	31.7 [25.3, 38.4]	31 [25.1,34.6]	13.9 [12.0, 17.1]
	Missing	7	21	20	0

a Median and the inter-quantile range are provided for each exposure, together with the number of missing observations.

b Measured in micrograms per milliliter whole blood.

c Measured in microgram per gram lipid.

d Σ3PCBs =(PCB138 + PCB153 + PCB180)*2.

e Total PFOS includes linear PFOS (L-PFOS, the most common form) and branched PFOS isomers, including 1,2,3,4, and 5 methylheptane sulfonate, iso-PFOS and other positional isomers.

**Table 3:** Association between PCBs and sperm disomy in single-exposure Poisson regression models with robust standard errors.^a^

Exposure	XX18	XY18	YY18	Total disomy
**Birth**				
PCB118	1.014^b^ [0.886;1.161]^c^	1.025 [0.932;1.127]	1.113 [0.930;1.331]	1.046 [0.940;1.164]
PCB138	1.148 [1.002;1.314]*	1.139 [1.023;1.268]*	1.232 [1.057;1.436]*	1.166 [1.055;1.288]*
PCB153	1.113 [0.945;1.312]	1.115 [0.989;1.258]	1.245 [1.033;1.501]*	1.150 [1.019;1.298]*
PCB180	1.016 [0.897;1.150]	1.073 [0.991;1.161]	1.087 [0.909;1.301]	1.063 [0.959;1.179]
Σ3PCBs^d^	1.121 [0.955;1.316]	1.136 [1.012;1.275]*	1.246 [1.038;1.495]*	1.163 [1.035;1.306]*
p,p´-DDE	1.030 [0.881;1.204]	1.042 [0.929;1.170]	1.127 [0.959;1.323]	1.063 [0.946;1.195]
**Age 7**				
PCB118	1.398 [1.067;1.833]*	1.052 [0.831;1.330]	1.082 [0.781;1.500]	1.127 [0.935;1.360]
PCB138	1.361 [1.050;1.766]*	1.065 [0.846;1.339]	1.081 [0.812;1.439]	1.129 [0.941;1.353]
PCB153	1.361 [1.072;1.729]*	1.103 [0.901;1.350]	1.147 [0.837;1.573]	1.167 [0.994;1.370]
PCB180	1.315 [1.035;1.672]*	1.063 [0.886;1.275]	1.116 [0.823;1.515]	1.129 [0.971;1.312]
3ΣPCBs	1.353 [1.056;1.735]*	1.081 [0.881;1.328]	1.123 [0.823;1.533]	1.148 [0.972;1.355]
p,p´-DDE	1.204 [1.023;1.418]*	1.056 [0.912;1.224]	1.197 [0.944;1.519]	1.125 [0.973;1.302]
**Age 14**				
PCB118	1.141 [0.955;1.362]	1.036 [0.873;1.231]	1.118 [0.912;1.372]	1.080 [0.930;1.254]
PCB138	1.160 [0.995;1.352]	1.048 [0.908;1.211]	1.138 [0.933;1.388]	1.095 [0.964;1.243]
PCB153	1.165 [1.004;1.352]*	1.048 [0.919;1.194]	1.113 [0.923;1.343]	1.089 [0.970;1.223]
PCB180	1.189 [1.053;1.342]*	1.086 [0.966;1.221]	1.144 [0.959;1.365]	1.123 [1.013;1.245]*
3ΣPCBs	1.188 [1.039;1.358]*	1.067 [0.937;1.215]	1.135 [0.938;1.373]	1.110 [0.991;1.243]
p,p´-DDE	0.986 [0.813;1.195]	0.901 [0.759;1.069]	1.029 [0.839;1.261]	0.951 [0.807;1.121]
**Age 22**				
PCB118	1.066 [0.944;1.203]	1.110 [1.000;1.233]	1.036 [0.907;1.183]	1.079 [0.983;1.185]
PCB138	1.090 [0.923;1.287]	1.116 [0.974;1.279]	1.099 [0.941;1.283]	1.106 [0.986;1.241]
PCB153	1.087 [0.912;1.296]	1.133 [0.988;1.299]	1.131 [0.941;1.359]	1.123 [0.994;1.268]
PCB180	1.091 [0.920;1.293]	1.111 [0.977;1.264]	1.141 [0.952;1.366]	1.116 [0.990;1.258]
3ΣPCBs	1.093 [0.917;1.303]	1.126 [0.981;1.292]	1.131 [0.947;1.350]	1.121 [0.992;1.266]
p,p´-DDE	0.972 [0.883;1.069]	1.070 [0.994;1.151]	0.954 [0.865;1.051]	1.010 [0.942;1.083]

a adjusted for smoking status and number of abstinence days before semen collection and using robust standard errors. Exposures were log2-transformed and the log-transformed number of nuclei was included as an offset in the analysis.

b estimated relative change in the percentage of each disomy at a doubling of the exposure for two individuals within the same smoking status and abstinence time group.

c 95% confidence interval.

* indicates whether the association is statistically significant (p-value < 0.05).

**Table 4:** Association between PFAS and sperm disomy in single-exposure Poisson regression models with robust standard errors.^a^

Exposure	XX18	XY18	YY18	Total disomy
***Birth***				
PFOA	0.935^b^ [0.743;1.177]^c^	0.934 [0.754;1.157]	0.950 [0.788;1.147]	0.938 [0.776;1.134]
PFOS^d^	1.375 [0.845;2.236]	1.029 [0.646;1.639]	1.019 [0.565;1.838]	1.094 [0.774;1.546]
**Age 7**				
PFOA	1.358 [0.720;2.561]	1.180 [0.619;2.248]	2.643 [1.153;6.058]*	1.522 [0.854;2.714]
PFOS	1.380 [0.779;2.443]	1.336 [0.723;2.469]	1.499 [0.808;2.779]	1.391 [0.832;2.327]
PFNA	1.602 [1.123;2.284]*	1.514 [1.053;2.177]*	1.688 [1.008;2.827]*	1.581 [1.190;2.100]*
PFHxS	1.335 [1.110;1.607]*	1.320 [1.133;1.538]*	1.363 [0.982;1.891]	1.335 [1.162;1.534]*
PFDA	1.398 [0.982;1.990]	1.350 [0.946;1.926]	1.425 [0.803;2.529]	1.382 [1.019;1.875]*
**Age 14**				
PFOA	0.602 [0.313;1.161]	0.556 [0.292;1.057]	0.827 [0.316;2.161]	0.627 [0.345;1.140]
PFOS	1.239 [0.416;3.691]	0.920 [0.309;2.738]	0.631 [0.284;1.402]	0.897 [0.367;2.190]
PFNA	1.574 [1.082;2.290]*	1.492 [1.010;2.205]*	0.739 [0.372;1.466]	1.281 [0.912;1.800]
PFHxS	1.878 [1.212;2.910]*	1.529 [1.004;2.328]*	0.984 [0.501;1.933]	1.430 [0.969;2.108]
PFDA	0.936 [0.518;1.692]	1.084 [0.651;1.806]	0.837 [0.524;1.338]	0.982 [0.645;1.494]
**Age 22**				
PFOA	0.949 [0.586;1.537]	1.053 [0.745;1.487]	0.999 [0.505;1.980]	1.014 [0.691;1.488]
PFOS	1.806 [1.030;3.166]*	1.309 [0.802;2.134]	1.346 [0.786;2.305]	1.414 [0.937;2.132]
PFNA	1.946 [1.180;3.210]*	1.608 [1.005;2.573]*	1.394 [0.776;2.504]	1.608 [1.077;2.400]*
PFHxS	1.880 [1.308;2.703]*	1.520 [1.017;2.270]*	1.243 [0.598;2.580]	1.502 [1.033;2.185]*
PFDA	1.350 [0.734;2.482]	1.396 [0.893;2.183]	1.346 [0.689;2.629]	1.371 [0.890;2.113]

a adjusted by smoking status and number of abstinence days before semen collection and using robust standard errors. Exposures were log2-transformed and the log-transformed number of nuclei was included as an offset in the analysis.

b estimated relative change in the percentage of each disomy at a doubling of the exposure for two individuals within the same smoking status and abstinence time group.

c 95% confidence interval.

d Results were not available for PFDA, PFNA and PFHxS in cord blood because the majority of values were undetectable in the sample.

* indicates whether the association is statistically significant (p-value < 0.05).

**Table 5:** Association of PCBs and PFASs and sperm disomy for mixture effect from quantile g-computation for Poisson regression models with robust standard errors.^a^

Analysis		XX18		XY18		YY18		Total disomy	
		Estimate^b^	P-value	Estimate	P-value	Estimate	P-value	Estimate	P-value
**PCBs** ^c^	Cord blood	1.161	0.082	1.124	0.069	1.285	0.006*	1.176	0.009*
	Age 7	1.323	0.018*	1.042	0.746	0.997	0.98	1.086	0.329
	Age 14	1.129	0.105	1.030	0.7	1.152	0.161	1.081	0.243
	Age 22	1.082	0.381	1.136	0.056	1.142	0.182	1.126	0.043*
**PFASs** ^d^	Cord blood	1.230	0.195	1.009	0.958	1.002	0.99	1.051	0.674
	Age 7	1.323	0.195	1.222	0.384	1.931	0.015*	1.416	0.083
	Age 14	1.331	0.399	0.945	0.864	0.677	0.333	0.949	0.859
	Age 22	1.653	0.025*	1.427	0.047*	1.292	0.39	1.429	0.043*
**PFASs/PCBs** ^e^	Cord blood	1.186	0.323	0.995	0.976	0.989	0.954	1.031	0.795
	Age 7	0.970	0.892	1.418	0.145	1.451	0.133	1.370	0.082
	Age 14	1.095	0.765	1.034	0.91	0.569	0.215	0.907	0.71
	Age 22	1.708	0.011*	1.382	0.057	1.175	0.522	1.378	0.042*
**PCBs/PFASs** ^f^	Cord blood	1.129	0.179	1.112	0.129	1.285	0.007*	1.162	0.022*
	Age 7	1.418	0.026*	1.062	0.685	1.001	0.994	1.122	0.389
	Age 14	0.994	0.951	0.879	0.082	1.157	0.207	0.971	0.684
	Age 22	0.981	0.845	1.085	0.207	1.093	0.4	1.065	0.341
**PCBs PFASs** ^g^	Cord blood	1.340	0.100	1.107	0.549	1.272	0.249	1.201	0.197
	Age 7	1.375	0.155	1.506	0.063	1.452	0.153	1.537	0.007*
	Age 14	1.089	0.781	0.909	0.747	0.659	0.338	0.880	0.639
	Age 22	1.675	0.008*	1.501	0.013*	1.284	0.328	1.467	0.009*

a Log-transformed exposures are included in the model adjusted by smoking status and days of abstinence.

b The estimate is the estimated relative change in percentage of disomy if all exposure concentrations in the mixture were doubled.

c mixture effect of PCBs (138,153,180).

d mixture effect of PFASs (PFOA, PFNA, PFHxS, tot PFOS ).

e mixture effect of PCBs when adjusting for PFASs.

f mixture effect of PFASs when adjusting for PCBs.

g mixture effect for PCBS and PFASs together.

* indicates whether the association is statistically significant (p-value < 0.05); p-values are for a two-sided test of a null mixture effect (score test).

Supplementary Table 1: Association of PCBs and PFASs and sperm disomy for mixture effect for quantile g-computation with unidirectionality assumption (non-negativity restriction) for a Poisson regression model with robust standard errors^a^

## Data Availability

The Faroe Island Cohort data are available to collaborating scientists following strict data privacy protocols, in accordance with the General Data Protocol Regulation (GDPR).
